# Analysing the Impacts of Financial Support for Regional Suicide Prevention Programmes on Suicide Mortality Caused by Major Suicide Motives in Japan Using Statistical Government Data

**DOI:** 10.3390/ijerph18073414

**Published:** 2021-03-25

**Authors:** Tomosuke Nakano, Toshiki Hasegawa, Motohiro Okada

**Affiliations:** Department of Neuropsychiatry, Division of Neuroscience, Graduate School of Medicine, Mie University, Tsu 514-8507, Japan; t-nakano@clin.medic.mie-u.ac.jp (T.N.); t-hasegawa@clin.medic.mie-u.ac.jp (T.H.)

**Keywords:** suicide mortality, suicide prevention, suicide motive, Japan, prefecture, municipality, financial support

## Abstract

To improve and plan regional suicide prevention programmes that utilise more cost-effective governmental financial support compared with previous programmes, the present study determined the effects of the amount of financial support provided for regional suicide prevention programmes, such as the Emergency Fund to Enhance Community-Based Suicide Countermeasures (EFECBSC), on the trends of suicide mortalities caused by six major suicide motives between 2009 and 2018, using forward multiple regression analysis. The ranking order of motives for male suicide was health, economy, family, employment, romance and school (in that order), whereas the ranking order for females was health, family, economy, romance, employment and school. Male suicide mortality caused by economy-related motives was significantly/inversely related to prefectural intervention programmes, whereas mortality caused by health-related motives was also significantly/inversely related to prefectural intervention programmes, but significantly/positively related to prefectural personal consultation support programmes. Contrary to males, female suicide mortality caused by health-related motives was significantly/inversely related to the municipal development programmes of leaders/listeners, whereas mortality caused by family- and school-related motives was significantly/positively related to prefectural and municipal telephone consultation support programmes, respectively. Contrary to our expectations, school-aged female suicide mortality caused by school-related motives was significantly/positively related to prefectural personal consultation support, enlightenment and municipal telephone consultation support programmes. These results indicate that Japanese regional suicide prevention programmes probably affect the suppression of male suicide mortality. However, these programmes are possibly ineffective, or at least partially, have an adverse effect, in regard to the suicide mortalities of female and school-aged populations. Therefore, we should work to improve regional suicide prevention programmes, making them more cost-effective and targeted towards female and school-aged populations in the future.

## 1. Introduction

The World Health Organization (WHO) estimates that there were over 800,000 suicide deaths worldwide in 2016, with an annual global age-standardised suicide mortality of 10.5 per 100,000 people [1,2,3]. Thus, suicide is one of the most frequent causes of death in worldwide, and notably in young individuals [1,2,3]. Globally, suicide mortality has recorded an increase of more than 50% in the last half century, with the increase in suicide mortality being more pronounced in adolescents [1,2,4]. Similarly, suicide mortality in Japan suddenly rose to more than 30,000 deaths in 1998 (at its maximum, there were 40.1 deaths per 100,000 males in 2003), and this increase in Japanese suicide mortality persisted for more than a decade (until 2009) [5,6,7,8]. Japanese people had been demanding government legislation and financial support for the development of national comprehensive suicide prevention programmes [7,8,9]. The Japanese government enacted the “Basic Act on Suicide Prevention” in 2006 and the “General Policies for Comprehensive Measures against Suicide” in 2007, but Japanese suicide mortality did not fluctuate for 3 years (until 2009) [7,8]. To respond to the Japanese public health crisis, the Cabinet Office began to contribute funds to prefectures and municipalities for the development of regional suicide prevention programmes, in the form of the “Emergency Fund to Enhance Community-Based Suicide Countermeasures” (EFECBSC), in 2009 [7,8,10,11,12]. Recent studies suggest that the EFECBSC played a fundamental role in the decline of the long-lasting high levels of suicide mortality in Japan [7,8].

The EFECBSC was composed of ten sub-divisions of regional support programmes with five independent prefectural and five municipal suicide prevention programmes [7,8,11]: (“personal consultation support programme”, “telephone consultation support programme”, “development programme of leaders/listeners”, “enlightenment programme” and “intervention model programme” [7,8,11]). Municipalities submitted their municipal suicide prevention programmes to their prefectures. Then, the prefecture submitted its prefectural programme with municipal programmes to the EFECBSC office in the Cabinet Office, which allocated funds to each prefecture, including budgets for prefectures and municipalities. These prefectural and municipal suicide prevention programmes were independent of each other [7,8,10,11]. Personal consultation support programmes support the development of a multidisciplinary support system and community support consultation sessions to combat economic problems (unemployment, bankruptcy and multiple debts) and health problems that are risk factors for suicide, as well as a system of broad collaboration between professionals, such as “lawyers, psychiatrists, social workers, and public health nurses” [7,8,10,11]. Telephone consultation support programmes support the enhancement of the telephone and internet support system implemented by the prefecture, municipality and private organisations (sharing of telephone numbers, free 24-h support, etc.) [7,8,10,11]. The development programmes of leaders/listeners organise workshops for human resources training to provide consultation support for persons at high risk of suicide, persons who have attempted suicide and bereaved family members (gatekeepers, leaders and listeners) [7,8,10,11]. The enlightenment programme provides financial support for public relations, such as newspapers, television, radio, pamphlets, symposiums or lectures, etc., in order to raise awareness of social and public support for high-risk people [8,9,10,11]. The intervention model programme offers survey and support programmes aimed at high-risk individuals that are independently implemented by prefectures and municipalities [7,8,10,11].

Although recent studies reported that the EFECBSC possibly played a fundamental role in the decline of the long-lasting high levels of suicide mortality in Japan [7,8], other studies also revealed that the response in relation to the suicide mortality of gender- and age-based subgroups did not distribute evenly across all EFECBSC sub-divisions [7,8]. As such, male suicide mortality was related significantly/inversely (decreased) to funding for the EFECBSC of both prefectures and municipalities, whereas female suicide mortality was related significantly/inversely to municipal funding amounts, but not those of prefectures [8]. Furthermore, the male suicide mortality of over 50s was possibly decreased by prefectural funding for the EFECBSC; however, the sensitivity of the suicide mortality of middle-/working-aged groups (20s, 30s and 40s) in regard to funding for the EFECBSC was less, meaning that the regional suicide prevention programmes cannot address the welfare/social safety nets and regional social protection vulnerability associated with suicide in working-age populations [7,8]. 

It has been well established that chronic exposure to various psychosocial distress plays an important role and is intricately intertwined with individuals’ intentions to commit suicide [13]. Based on understanding the suicide motives associated with complicated psychosocial distress/risk factors, effective suicide prevention programmes targeting critical suicide motives should lead to a reduction in suicide mortality. Several psychosocial distress/risk factors, including hopelessness, psych-ache, impulsivity and negative life events, have been explored in attempts to predict suicide motives [14,15,16]. It has been suggested that negative future expectancy plays major roles in suicide motives, such as hopelessness, leading to secondary affective disturbance [17,18]. It has been considered that the Japanese sociocultural/psychosocial view of the self, which is predominantly dependent on relationships with other persons (compared to Western countries), probably influences future expectancy/hopelessness [19]. Therefore, chronic exposure to psychosocial distress plays a more important role in suicide motives in Japan compared with Western countries [20]. Based on the above background, the present study determines the effects of the ECEFBSC sub-divisions on suicide mortality caused by six major motives in order to develop more cost-effective and evidence-based regional suicide prevention programmes in Japan, according to the “Revised Basic Act on Suicide Prevention” of 2017 and the “Law Concerning the Promotion of Research and Utilisation of Results to Contribute to the Comprehensive and Effective Implementation of Suicide Prevention” of 2019 [6,10,11,12,21,22].

## 2. Materials and Methods

### 2.1. Data

Data regarding the complete unemployment rates (CUR) in each prefecture from 2009 to 2018 were derived from the Statistics Bureau of the Ministry of Internal Affairs and Communications (SBMIAC) [23]. Suicide motives, age-, gender- and prefecture-disaggregated numbers of suicides in all 47 prefectures of Japan between 2009 and 2018 were obtained from the “Basic Data on Suicide in the Region” (BDSR) of the Ministry of Health, Labour and Welfare (MHLW) and SBMIAC [6,24]. BDSR data were classified by the number of suicides into six types of suicide motives associated with health, family, economy, romance, employment and school [6]. In particular, the BDSR counts the number of suicide individuals in each region under the jurisdiction of local police stations. The police investigate personal characteristics and background factors of each suicide individual. The results of this investigation contain a number of motives for suicide, and these motives are compared to previously compiled suicide motives lists. Finally, the investigation identifies the possible motive for suicide, based on the evidence, suicide note or other documentation, such as medical certificate, clinical recording and testimony of surviving family [13].

Annual suicide motives-, gender- and prefecture-disaggregated suicide mortalities were derived from the BDSR, and population exposure (denominator) was obtained from the SBMIAC [24]. The BDSR publishes the number of suicide motives disaggregated by gender, but does not indicate the number of suicide motives disaggregated by age [6]. In particular, to analyse the effects of the EFECBSC on suicide mortality caused by school-related motives, the suicide mortalities caused by school-related motives were calculated by setting two types of denominators—the whole population (similar to the other suicide motives) and the school-aged population (younger than 20 years old)—since it was speculated that suicide caused by school-related motives was possibly a specific factor of school-aged individuals. The funding for the 10 sub-divisions of EFECBSC between 2009 and 2014 [11] of each prefecture was calculated by dividing the EFECBSC for each year by the prefectural population of the same year. 

The sum of male plus female (Male+Female) and separate male and female suicide mortalities caused by six motives were calculated. All 47 prefectures have a large population distribution, with mean ±SD = 2.7 ± 2.6 million, median = 1.7 million, maximum = 12.5 million, and minimum = 0.6 million. Therefore, to eliminate small population artefacts, gender-disaggregated prefectural suicide mortalities caused by suicide motives were calculated using the empirical Bayes standardised mobile ratio (EBSMR) [25] using the EB estimator for the Poisson/gamma model version 2.1 (National Institute of Public Health, Wako, Japan) (https://www.niph.go.jp/soshiki/gijutsu/download/ebpoig/index_j.html accessed on 1 March 2021) [7,8]. 

### 2.2. Statistical Analysis

EFECBSC, which was established as a fund rather than a grant, was not exhausted by the end of the year but was allocated to be executed within the EFECBSC period [7,8,11]. The least squares method was used to analyse time-dependent trends in the changes in suicide EBSMR in each prefecture (EBSMR trends) using the free statistical software HAD version 17 (Shimuzu, H., Kwansei Gakuin University, Nishinomiya, Hyogo) (https://osf.io/32cyp/files/, accessed on 1 March 2021) [26,27]. To analyse the relationship between EBSMR trends caused by six major motives, CUR and financial support for regional prevention programmes (funding for the EFECBSC sub-divisions), SPSS for Windows version 27 (IBM, Armonk, NY, USA) was used for the forward multiple regression analyses, in order to analyse the effects of CUR trends and funding for the 10 EFECBSC sub-divisions on EBSMR trends. Multicollinearity was suspected if the variance inflation factor (VIF) value was greater than 10 [7,8]. The relationship between the suicide mortality of the school-aged population (younger than 20 years old) and suicide mortality caused by school-related motives in school-aged populations was analysed using linear regression analysis (SPSS). The time-dependent reduction in EBSMR caused by suicide motives was analysed by linear mixed model analysis (SPSS) followed by Tukey’s post hoc test using BellCurve for Excel, version 3.2 (BellCurve, Tokyo, Japan), when the F-value of the year factor was significant (*p* < 0.05) [28,29,30]. 

## 3. Results

### 3.1. EBSMR Trends of Regional Suicide Mortality Associated with Six Suicide Motives between 2009 and 2018

**Figure 1:**
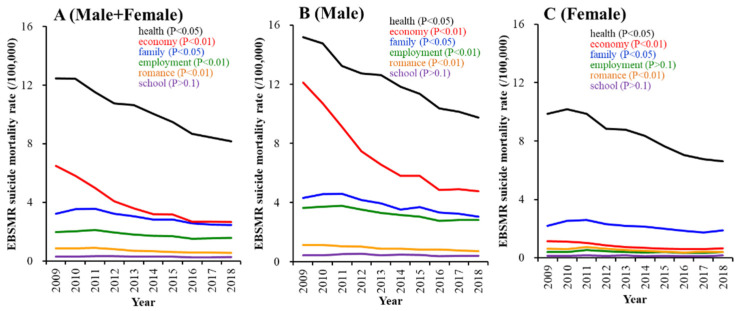
Trends of empirical Bayes standardised mobile ratios (EBSMRs) of suicide mortality in the Japanese population per 100,000 population in males plus females (Male+Female) (**A**), males (**B**) and females (**C**) between 2009 and 2018 were analysed by linear mixed model analysis. Ordinates indicate EBSMR (per 100,000 population), and abscissas indicate time (year). The F-values of the linear mixed model analysis were F_motive_ (5, 119) = 4953.6 (*p* < 0.01), F_gender_ (1, 119) = 0.0 (*p* > 0.1), F_year_ (9, 119) = 0.0 (*p* > 0.1), F_motive*gender_ (5, 119) = 610.7 (*p* < 0.01), F_motive*year_ (45, 119) = 2.0 (*p* < 0.01) and F_gender*year_ (9, 119) = 0.0 (*p* > 0.1).

In 2009, the top six motives for suicide ranked in order in Male+Female (sum of male plus female) and males alone were health, economy, family, employment, romance and school (in that order), whereas the ranking order for females alone was health, family, economy, romance, employment and school [12]. The indicated EBSMR rankings of suicide motives in Male+Female, males and females were consistent over a decade between 2009 and 2018 (Figure 1). 

Linear mixed model analysis detected the differences between male and female suicide mortalities caused by motives associated with health, economy, family and employment, but male and female EBSMRs caused by romance- and school-related motives were almost equal. The female EBSMR of suicide mortalities caused by health- and family-related motives was larger than that of males at 66~69% vs. 41~46% and 15~19% vs. 12~15%, respectively, whereas the male EBSMR of suicide mortalities caused by economy- and employment-related motives was larger than that of females at 22~33% vs. 6~8% and 10~13% vs. 3~4%, respectively (Table 1).

The EBSMRs of suicide mortalities caused by health-, economy-, family-, employment- and romance-related motives of Male+Female and males were significantly decreased in a time-dependent manner (*p* < 0.05), but those caused by school-related motives in Male+Female and males were unchanged (Figure 1A,B). Female EBSMRs of suicide mortality caused by health-, economy-, family- and romance-related motives were significantly decreased, in a time-dependent manner (*p* < 0.05), but neither those caused by employment- nor school-related motives in females were changed (Figure 1C). 

### 3.2. Effects of Funding Amounts Provided to EFECBSC Sub-Divisions on EBSMR Trends of Suicide Mortalities Caused by Six Suicide Motives between 2009 and 2018

Forward multiple regression analysis detected significant effects of funding for the EFECBSC sub-divisions on EBSMR trends of suicide mortalities caused by family-, health-, economy- and school-related motives, whereas neither EBSMR trends of suicide mortalities caused by employment- nor romance-related motives were affected by EFECBSC. The detailed statistical values of the forward multiple regression analysis are shown in Table 2 (data for excluded variables are provided in Supplementary Tables S1–S4).

#### 3.2.1. Effects of Amounts of Funding Provided to EFECBSC Sub-Divisions on EBSMR Trends of Suicide Mortalities Caused by Health-Related Motive between 2009 and 2018

**Figure 2:**
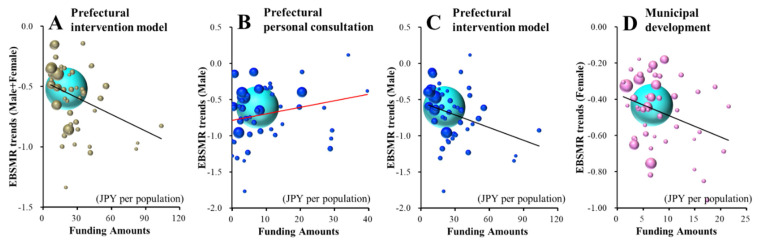
Effects of the amount of funding provided to sub-divisions of EFECBSC on EBSMR trends of Male+Female (**A**), male (**B**,**C**) and female (**D**) suicide mortalities caused by health-related motives were analysed by forward multiple regression analysis. Ordinates indicate EBSMR trends of suicide mortality caused by health-related motives between 2009 and 2018, and abscissas indicate the amount of funding provided to EFECBSC sub-divisions (JPY per population). Light blue, grey, blue and red spheres indicate national, Male+Female, male and female EBSMR trends and population sizes, respectively. Black and red lines indicate the regression lines of significantly positive (decreases suicide mortality) and negative (increases suicide mortality) factors, respectively. Male+Female EBSMR trends of suicide mortality caused by health-related motives = −0.0046 × (prefectural intervention model programme) −0.455. Male EBSMR trends of suicide caused by health-related motives = −0.0067 × (prefectural intervention model programme) + 0.0113 × (prefectural personal consultation support programme) − 0.624. Female EBSMR trends of suicide caused by health-related motives = −0.0118 × (municipal development programme of leaders/listeners) − 0.372.

Male+Female EBSMR trends of suicide mortality caused by health-related motives were significantly/inversely related to funding for the prefectural intervention model programme (Figure 2A and Table 2). The multiple regression analysis results indicated that JPY 21.7 million per 100,000 population for the prefectural intervention model programme decreases the Male+Female EBSMR of suicide mortality caused by health-related motives by one per year. The male EBSMR trends of suicide mortality caused by health-related motives were significantly/inversely related to the funding of the prefectural intervention model programme, but were significantly/positively related to that of the prefectural personal consultation support programme (Figure 2B,C and Table 2). JPY 15.0 million per 100,000 population for the prefectural intervention model programme and JPY 8.9 million per 100,000 population for the prefectural personal consultation support programme decreases and increases the male EBSMR of suicide mortality caused by health-related motives by one per year, respectively.

The female EBSMRs of suicide mortality caused by health-related motives were significantly/inversely related to the amount of funding provided to the municipal development programme of leaders/listeners (Table 2 and Figure 2D). JPY 8.5 million per 100,000 population for the municipal development programme of leaders/listeners decreases the female EBSMR of suicide mortality caused by health-related motives by one per year.

#### 3.2.2. Effects of Amounts of Funding Provided to EFECBSC Sub-Divisions on EBSMR Trends of Suicide Caused by Economy-Related Motives between 2009 and 2018

**Figure 3:**
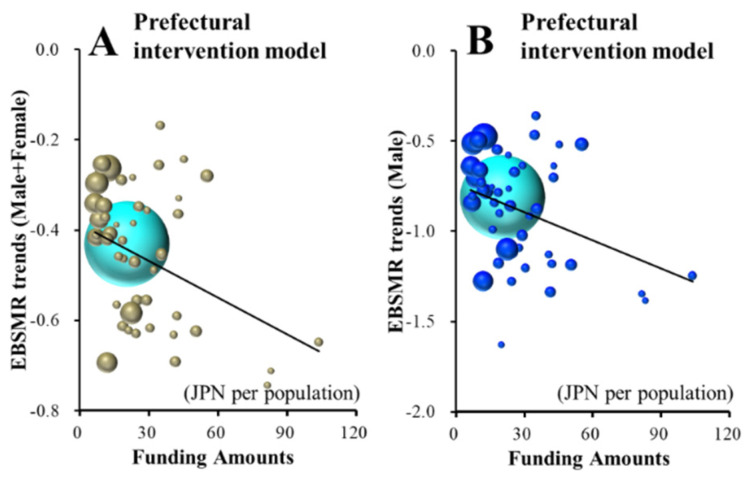
Effects of the amount of funding provided to sub-divisions of EFECBSC on EBSMR trends of Male + Female (**A**) and male (**B**) suicide mortalities caused by economy-related motive were analysed by forward multiple regression analysis. Ordinates indicate EBSMR trends of suicide mortality caused by health-related motives between 2009 and 2018, and abscissas indicate the amount of funding provided to EFECBSC sub-divisions (JPY per population). Light blue, grey and blue spheres indicate national, Male+Female and male EBSMR trends and population sizes, respectively. Black lines indicate the regression lines of significantly positive (decreases suicide mortality) factors. Male+Female EBSMR trends of suicide caused by economy-related motives = −0.0027 × (prefectural intervention model programme) − 0.388. Male EBSMR trends of suicide caused by economy-related motives = −0.0052 × (prefectural intervention model programme) − 0.738.

Both Male+Female and male EBSMR trends of suicide mortalities caused by economy-related motives were significantly/inversely related to the amount of funding provided to the prefectural intervention model programme (Figure 3A,B and Table 2). Female EBSMR trends of suicide mortality caused by economy-related motives were not related to any amount of funding for the EFECBSC sub-divisions (Supplementary Table S2). Multiple regression analysis results indicated that JPY 37.0 million and JPY 19.20 million per 100,000 population for the prefectural intervention model programme decrease Male+Female and male EBSMRs of suicide mortality caused by economy-related motives by one per year, respectively.

#### 3.2.3. Effects of Amounts of Funding Provided to EFECBSC Sub-Divisions on EBSMR Trends of Suicide Caused by Family- and School-Related Motives between 2009 and 2018

**Figure 4:**
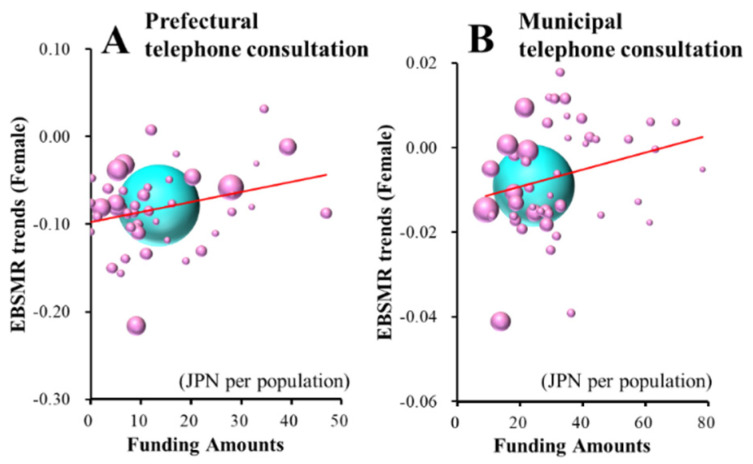
Effects of the amount of funding provided to sub-divisions of EFECBSC on female EBSMR trends of suicide mortalities caused by family- (**A**) and school-related (**B**) motives were analysed by forward multiple regression analysis. Ordinates indicate EBSMR trends of suicide mortality caused by family- and school-related motives between 2009 and 2018, and abscissas indicate the amount of funding provided to EFECBSC sub-divisions (JPY per population). Light blue and red spheres indicate national and female EBSMR trends and population sizes, respectively. Red lines indicate the regression lines of significantly negative (increases suicide mortality) factors. Female EBSMR trends of suicide caused by family-related motive = 0.0011 × (prefectural telephone consultation support programme) − 0.098. Female EBSMR trends of suicide caused by school-related motive = 0.0010 × (municipal telephone consultation support programme) − 0.004.

Neither Male+Female nor male EBSMR trends of suicide mortalities caused by family- and school-related motives were affected by any amount of funding for the EFECBSC sub-divisions (Supplementary Tables S3 and S4). Contrarily, female EBSMR trends of suicide mortalities caused by family- and school-related motives were significantly/positively related to prefectural and municipal telephone consultation support programmes, respectively (Figure 4A,B and Table 2). JPY 87.3 million and JPY 156.0 million per 100,000 population for prefectural and municipal telephone consultation support programmes increase female EBSMRs of suicide mortality caused by family- and school-related motives by one per year, respectively.

### 3.3. Effects of Amounts of Funding Provided to EFECBSC Sub-Divisions on EBSMR Trends of School-Aged Population between 2009 and 2018

**Figure 5:**
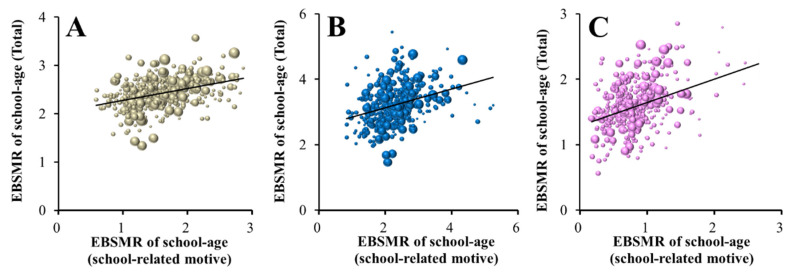
Relationships between EBSMRs of suicide mortalities of school-aged population (total) (younger than 20 years old) and suicide mortalities caused by school-related motives of school-aged populations in Male+Female (**A**), males (**B**) and females (**C**) were analysed by linear regression analysis. Abscissas indicate the EBSMRs of school-related motives among those over 10 years old, and ordinates indicate the EBSMRs of the suicide numbers of those over 10 years old between 2009 and 2018. Grey, blue and red spheres indicate Male+Female, male and female EBSMRs of all prefectures from 2009 to 2018. Black lines indicate the regression lines of significantly positive factors.

Neither Male+Female, male nor female EBSMR suicide mortalities caused by school-related motives changed between 2009 and 2018 (Figure 1); however, contrary to our expectation, female EBSMR suicide mortality caused by school-related motives was positively related to the municipal telephone consultation support programme (Figure 4B). School-related problems are a possible dominant motive for suicide in school-aged individuals. The BDSR does not publish the age-disaggregated suicide numbers caused by each motive [6]. Therefore, the relationship between the Male+Female, male and female EBSMRs of the school-aged population (total: suicide numbers of those younger than 20 years old/population of those younger than 20 years old) and estimated EBSMRs of suicide mortality caused by school-related motives of school-aged populations (school-related motive: suicide numbers caused by school-related motives/population of those younger than 20 years old) was analysed by linear regression analysis. The Male+Female, male and female EBSMRs of school-age (total) were significantly/positively related to the respective Male+Female (r^2^ = 0.37, *p* < 0.01), male (r^2^ = 0.33, *p* < 0.01) and female (r^2^ = 0.43, *p* < 0.01) EBSMRs of suicide mortality caused by school-related motives of school-age populations (school-related motive) (Figure 5A–C). 

These results suggest that school-related motives probably play an important role in the suicides of school-aged individuals. Therefore, the effects of the EFECBSC sub-divisions on the EBSMRs of suicide mortality among those over 10 years old and school-related motives among those over 10 years old were analysed by forward multiple regression analysis. Forward multiple regression analysis detected significant effects of funding for the EFECBSC sub-divisions on EBSMR trends among those over 10 years old in relation to suicide mortality and EBSMRs for suicides caused by school-related motives among those over 10 years old. The detailed statistical values of the forward multiple regression analysis are presented in Table 3 (excluded variable data are presented in Supplementary Tables S5 and S6).

#### 3.3.1. Effects of Amounts of Funding Provided to EFECBSC Sub-Divisions on Male+Female and Male EBSMR Trends of School-Aged Population and Caused by School-Related Motive between 2009 and 2018

**Figure 6:**
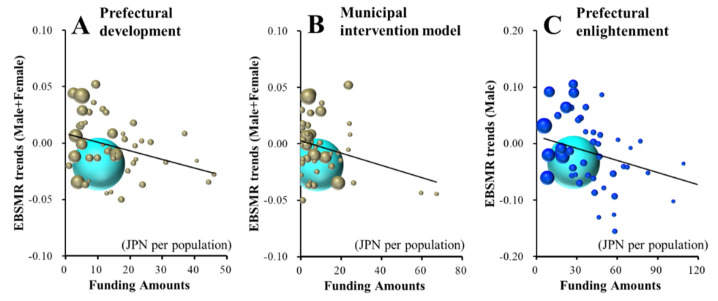
Effects of the amount of funding provided to sub-divisions of EFECBSC on the EBSMR trends of Male+Female (**A**,**B**) and male (**C**) suicide mortalities of school-aged population were analysed by forward multiple regression analysis. Ordinates indicate EBSMR trends for suicide mortality among those over 10 years old between 2009 and 2018, and abscissas indicate the amount of funding provided to EFECBSC sub-divisions (JPY per population). Light blue, grey and blue spheres indicate national, Male+Female and male EBSMR trends and population sizes, respectively. Black lines indicate the regression lines of significantly positive (decreases suicide mortality) factors. Male+Female EBSMR trends of school-aged populations = −0.0008 × (prefectural development programme of leaders/listeners) − 0.0006 × (municipal intervention model programme) + 0.015. Male EBSMR trends of school-aged populations = −0.0006 × (prefectural enlightenment programme) + 0.029.

Male+Female EBSMR trends of suicide mortality of school-aged population (total: younger than 20 years old) were significantly/inversely related to the amount of funding provided to the prefectural development programme of leaders/listeners and the municipal intervention model programme (Figure 6A,B and Table 3), whereas Male+Female EBSMR trends of suicide mortality caused by school-related motives of the school-aged population (school-related motive) were not related to the funding provided to EFECBSC sub-divisions (Supplementary Table S6). JPY 124.5 million per 100,000 population for the prefectural development programme of leaders/listeners and JPY 176.7 million per 100,000 population for the municipal intervention model programme decrease the Male+Female EBSMRs of suicide mortality of school-aged populations by one per year.

Male EBSMR trends of suicide mortality of the school-aged population were significantly/inversely related to the amount of funding provided to the prefectural enlightenment programme (Figure 6C and Table 3), whereas male EBSMR trends of suicide mortality caused by school-related motives of school-aged populations were not related to any amount of funding provided to the EFECBSC sub-divisions (Supplementary Table S6). JPY 174.8 million per 100,000 population for the prefectural enlightenment programme decreases the male EBSMRs of suicide mortality of school-aged populations by one per year. 

#### 3.3.2. Effects of Amounts of Funding Provided to EFECBSC Sub-Divisions on Female EBSMR Trends of School-Aged Population and Caused by School-Related Motive between 2009 and 2018

**Figure 7:**
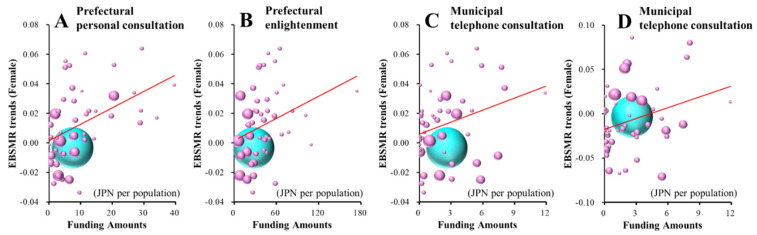
Effects of the amount of funding provided to sub-divisions of EFECBSC on female EBSMR trends of suicide mortalities of school-aged populations (**A**–**C**) and caused by school-related motives of school-aged populations (**D**) were analysed by forward multiple regression analysis. Ordinates indicate female EBSMR trends of suicide mortality of school-aged populations and school-related motives of school-aged populations between 2009 and 2018, and abscissas indicate the amount of funding provided to EFECBSC sub-divisions (JPY per population). Light blue and red spheres indicate national and female EBSMR trends and population sizes, respectively. Red lines indicate the regression lines of significantly negative (increased suicide mortality) factors. Female EBSMR trends of school-aged populations = 0.0009 × (prefectural personal consultation support programme) + 0.0003 (prefectural enlightenment programme) + 0.0030 (municipal telephone consultation support programme) − 0.0145. Female EBSMR trends of suicide mortality of school-related motives of school-aged populations = 0.0041 × (municipal telephone consultation support programme) − 0.180.

Female EBSMR trends of suicide mortality of school-aged populations were significantly/positively related to funding for the prefectural personal consultation support, enlightenment and municipal telephone consultation support programmes (Figure 7A–C and Table 3). Female EBSMR trends of suicide mortality caused by school-related motives of school-aged populations were significantly/positively related to the amount of funding provided to the municipal telephone consultation support programme (Figure 7D and Table 3). JPY 106.6 million, JPY 400.0 million and JPY 33.8 million per 100,000 population for prefectural personal consultation support, enlightenment and municipal telephone support programmes increase the female EBSMRs of suicide mortality of school-aged populations by one per year, respectively. JPY 24.2 million per 100,000 population for the municipal telephone consultation support programme increases the female EBSMRs of suicide mortality of school-related motives of school-aged populations.

## 4. Discussion

Numerous reports have identified economic strain as a cause for suicide [20,31]; however, the kinetics of suicide mortality in Japan are inconsistent with economic strain. Until the early 1990s, Japanese male suicide mortality was lower than that of European males [7,8]. Even after the asset bubble in 1991 (the largest Japanese economic crisis), Japanese suicide mortality did not significantly increase, but a drastic elevation of suicide mortality was recorded in 1998 and this high rate of suicide mortality among the Japanese population remained continuous for one decade (at its maximum, there were 40.1 deaths per 100,000 males in 2003). Notably, Japanese suicide mortality has seen a continuous reduction since 2009, despite various economic indicators deteriorating during the global economic crisis triggered by the bankruptcy of the Lehman Brothers in 2008 [7,8]. Based on the “Basic Act on Suicide Prevention” of 2006 and the “General Policies for Comprehensive Measures against Suicide” of 2007, in 2009, the Japanese government started contributing to the EFECBSC in prefectures and municipalities to encourage the development of comprehensive regional suicide prevention programmes in the form of the EFECBSC, resulting in governmental contributions increasing by 100 times compared with previous contributions associated with suicide prevention [10,11,32]. Due to the rigid financial situation of the Japanese government, EFECBSC was allocated to prefectures and municipalities between 2009 and 2014 as a fund rather than a grant [10,11]. 

Recently, several reports have emphasised the impact of governmental financial support on the reduction in suicide mortality. An increase in participation in the Supplemental Nutrition Assistance Program (SNAP) in the USA decreased Male+Female and male suicide mortalities [33]. The impact of unemployment rates on suicide mortality was small, but the enhancement of labour market programme supports by governmental finances in Japan, Europe and USA protected socioeconomic disability-induced suicide mortality [7,8,34,35,36]. Furthermore, policies aimed at providing support/assistance to low-income individuals, income growth and divorce prevention are probably more effective for suicide prevention compared to mental health expenditures [37]. Similar to the aforementioned studies, the present study also detected an inverse relationship between the prevention effects of governmental financial support (EFECBSC) and Japanese suicide mortality. In response to general/global concerns posed by suicide, a number of research efforts have aimed to identify the theoretical risk factors of suicide in the last several decades. In spite of these efforts, the studies described above suggest that a dissociation analysis between suicide risks and suicide prevention factors can indicate possible useful findings in the planning of more cost-effective suicide prevention programmes. 

**Figure 8:**
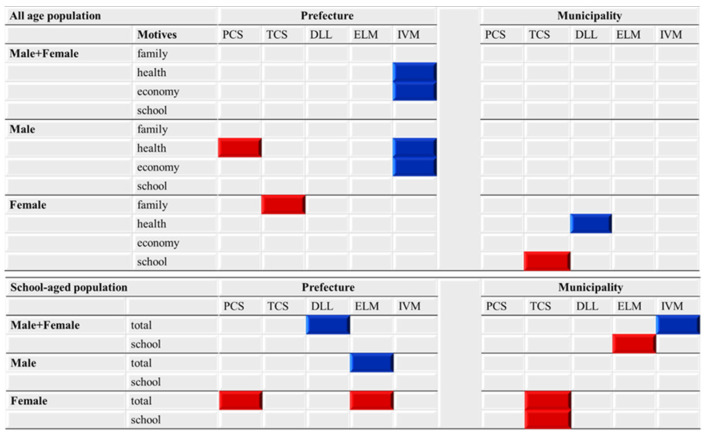
Summary of effects of the amount of funding provided to EFECBSC sub-divisions on Male+Female, male and female EBSMR trends of suicide mortalities caused by family-, health-, economy- and school-related motives. Blue and red columns indicate significant factors for reduced and increased EBSMR trends caused by each motive, detected by forward multiple regression analysis. DLL, development programme of leaders/listeners; ELM, enlightenment programme; IVM, intervention model programme; PCS, personal consultation support programme; TCS, telephone consultation support programme.

The present study demonstrated the inverse relation between the amount of funding provided to EFECBSC sub-divisions and suicide mortalities caused by specific motives, since the response of suicide mortalities caused by the six major subgroups of suicide motives (family-, health-, economy-, employment-, romance- and school-related motives) was not distributed evenly across the 10 EFECBSC sub-divisions. A summary of the effects of EFECBSC sub-divisions on EBSMR trends is provided in Figure 8.

In the present study, suicide mortalities of Male+Female, males and females caused by family-, health-, economy- or school-related motives were associated with funding for the EFECBSC (Figure 8). In particular, male suicide mortalities caused by health- and economy-related motives were specifically affected by prefectural suicide prevention programmes, whereas female suicide mortalities caused by health- and school-related motives were specifically affected by municipal prevention programmes (Figure 8). A discrepancy in the responses between male and female suicide mortalities when compared to EFECBSC sub-divisions was observed in a previous study, in which male suicide mortality was predominantly inversely related to prefectural suicide prevention programmes, whereas female suicide mortality was inversely related to municipal prevention programmes but was not affected by prefectural programmes [8]. 

Contrary to EFECBSC, CUR affected neither Male+Female, male nor female suicide mortalities caused by any suicide motives. The lack of an impact by CUR on any suicide mortality sub-categories caused by any motives was unexpected for us, since it has been generally established that complete unemployment should negatively affect the economic, familial and employment situations of individuals. Furthermore, a previous study reported that CUR significantly increased the suicide mortalities of male individuals over 50 years of age [7]. Therefore, CUR must contribute to suicide mortality as a suicide risk factor, specifically in (elder) male individuals. Unfortunately, the present study could not analyse the effects of CUR on age-dependent suicide mortalities caused by each suicide motive, since the BDSR does not publish detailed sub-categories of age-disaggregated suicide numbers caused by each motive [6]. In another unexpected finding, female suicide mortality caused by school-related motives was positively related to the municipal telephone consultation support programme. It has been established that the telephone consultation support programme is widely implemented for mental support and suicide prevention among young individuals in various countries, including Japan [38,39,40,41].

### 4.1. Effects of Amounts of Funding Provided to EFECBSC Sub-Divisions on Suicide Mortalities Caused by Suicide Motives between 2009 and 2018

Suicide mortalities caused by health-related motives were most frequent in male (41~46%) and female (66~69%) suicide mortalities between 2009 and 2018 in Japan. The reduction in both male and female numbers of suicides caused by health-related motives has contributed significantly to the decrease in suicide mortality in Japan, whereas the sensitivities between male and female suicide mortalities caused by health-related motives to EFECBSC sub-divisions are distinctly different. Indeed, female suicide mortality caused by health-related motives is inversely related to the amount of funding provided to the municipal development programme of leaders/listeners, whereas male suicide mortality caused by health-related motives is inversely and positively related to the amounts of funding provided to the prefectural intervention model and personal consultation support programmes. The BDSR does not publish detailed sub-categories of health-related motives [6]. However, a population-based study provided detailed sub-categories of gender-disaggregated suicide numbers caused by health-related motives using the database of the Ibaraki Prefectural Police Headquarters [13]. We analysed the suicide numbers caused by sub-categories of gender-disaggregated health-related motives in the database of the Ibaraki Prefectural Police Headquarters [13] using X^2^ analysis. The suicide mortality caused by a physical illness in males (38%) was larger than that of females (32%), whereas suicide mortalities caused by mental illness (65%) and major depression (46%) in females were larger than those caused by mental illness (57%) and major depression (38%) in males. Additionally, the preliminary suicide prevention programmes, prior to the “Basic Act on Suicide Prevention”, reported that the activation of social support resources by the municipal personal consultation support, development of leaders/listeners and intervention model programmes decreased female suicide mortality [42,43,44,45,46]. Females have a lower threshold when it comes to social/municipal resources and group meetings than males, resulting in a larger response by females to the municipal development programme of leaders/listeners [47]. Indeed, a recent study revealed that the municipal development programme of leaders/listeners resulted in a pronounced decrease in elder female suicide mortality specifically [7]. Taken together, alongside previous studies, the present study suggests that the Japanese regional suicide prevention programme has probably suppressed social integration disabilities among of females via community-based gatekeeping actions using the municipal development programme of leaders/listeners (but not the more extended prefectural development programme of leaders/listeners), resulting in a reduction in female suicide caused by mental illness.

Contrary to females, male suicide mortality caused by health-related motives was decreased by the prefectural intervention model programme, but increased by the prefectural personal consultation support programme. The effectiveness of the prefectural intervention model programme when it comes to male suicide mortality caused by health-related motives also suggests that males possibly require more active individual intervention due to the psychosocial features resistant to the community of males [7,8,47]. The negative effects of the prefectural personal consultation support programme on male suicide mortality caused by health-related motives must be cautiously discussed, since the personal consultation support programme supports the development of a multidisciplinary support system and community support consultation sessions to combat economic problems (unemployment, bankruptcy and multiple debts) and health problems that are risk factors for suicide, as well as a system of broad collaboration between professionals such as lawyers, psychiatrists, social workers, and public health nurses [7,8,10,11]. In spite of these efforts, our previous study reported that funding for the prefectural personal consultation support programme significantly increased suicide mortalities among elderly males [7,8]. We cannot conclude that the prefectural personal consultation support programme directly increased male suicide mortality caused by health-related motives; however, we can emphasise the two crucial disadvantages of the personal consultation support programme. The first is that the major targets of the personal consultation support programme are events that are difficult to solve, and it is necessary to spend an enormous amount on the construction of a multidisciplinary system of cooperation, as well as a large amount of time in order to accumulate experience. The other is that even if an effective personal consultation support programme is developed, the regional personal consultation support programme alone possibly does decrease the number of male individuals at a high risk of suicide. Our second belief is supported by the results that suicide mortality caused by economy-related motives (the major target of personal consultation support programme) was decreased by the prefectural intervention model programme, but unaffected by the prefectural personal consultation support programme. Thus, in order to develop more effective suicide prevention programmes, a systemised series of action procedures is a fundamental requirement: the detection of individuals at a high risk of suicide, the provision of guidance to help them access appropriate services and, finally, the development of rational solutions to the problems faced by such individuals. In other words, an effective suicide prevention programme is organised to complement the regional welfare and social safety nets that lead to an improvement in the vulnerability of regional social protection against suicidal behaviour/ideation via effective leading/exposure procedures, such as the regional intervention model programme. It is well known that patients with chronic physical disease, such as cancer [48], and chronic diseases, such as atopic dermatitis [49], asthma [50], migraine [51] and end-stage kidney disease treated with dialysis [52], are at a high risk of suicide and comorbidities with affective disturbance. Therefore, suicide mortality caused by physical illness-related motives could probably be prevented by the external cooperation of regional primary care physicians and psychiatry specialists, via suicide prevention programmes, due to the guidance to help them access appropriate services.

Female suicide mortality caused by family-related motives was increased by the municipal telephone consultation support programme, but male suicide mortality caused by family-related motives was not related to any EFECBSCs. In the data from the Ibaraki Prefectural Police Headquarters regarding suicide numbers caused by family-related motives [13], the major family-related motives were “Conflict with family members”, “Death of family member”, “Hopeless situation for the family” and “Exhaustion from support for family member”. In these four categories, female suicide caused by “Exhaustion from support for family member” was statistically larger than that of male suicide alone (20% vs. 5%). “Exhaustion from support for family member” comprised “caring for infirm family member(s)” (12%) and “raising children” (8%) [13]. In Japan, females continue to take primary responsibility for childcare in a social system that offers poor childcare support. Most women are forced to play subordinate roles due to the limited resources for childcare support and are prone to social isolation due to childcare demands [7,8]. Taken together with the results of a population-based study [13], the present study suggests that not only childcare, but also care of infirm family members contribute to the major suicide motives among females. Therefore, the telephone consultation support programme alone is probably insufficient to resolve the problems associated with childcare and caring for infirm family members. In other words, preventing female suicide mortality caused by childcare/caring for infirm family members possibly requires the development of community-based regional social welfare support programmes, rather than regional suicide prevention programmes, in Japan. 

### 4.2. Effects of Amounts of Funding Provided to EFECBSC Sub-Divisions on Suicide Mortalities of School-Aged Individuals between 2009 and 2018

It is a great surprise that the municipal telephone consultation support programme was significantly/positively related to female suicide mortality caused by school-related motives between 2009 and 2018, even though there were no statistically significant changes. School-related problems are potentially dominant motives for suicide in school-aged individuals. Thus, in order to analyse the effects of EFECBSC on suicide mortality caused by school-related motives in a more realistic way, the denominator used to calculate suicide mortality was changed from the whole population to the school-aged population (younger than 20 years old). The forward multiple regression analysis results for the relationship between the female EBSMR trends of suicide mortality of school-aged populations and those caused by school-related motives of school-aged populations were similar, but those of Male+Female and males were not affected by any EFECBSC sub-divisions (Figure 8). To explore the impacts of school-related motives on suicide mortality in school-aged individuals, the present study compared the effects of EFECBSC on suicide mortalities between EBSMR trends of suicide mortality of school-aged populations (total) and those caused by school-related motives (school-related motive).

The responsiveness of male suicide mortalities between total and school-related motives among school-aged populations was not detected consistently, since male suicide mortality of school-aged populations was inversely related to prefectural enlightenment, but male suicide mortality due to school-related motives of school-aged populations was not related to any EFECBSC sub-divisions. Contrary to male mortality, female suicide mortality due to school-related motives among school-aged populations was positively related to the municipal telephone consultation support programme, but female suicide mortality of school-aged populations was also positively related to the prefectural personal consultation support, enlightenment, and municipal telephone consultation support programmes. Therefore, the suicide of school-aged females, at least partially, composed of telephone consultation support programme-resistant suicide caused by school-related motives. Telephone consultation support programmes (also known as helplines, hotlines, crisis lines, etc.) are widely implemented to assist with a variety of psychosocial concerns among young people in a confidential, accessible format, such as via telephone, e-mail, social networking service or chatrooms [40,53]. Regarding young individuals, a global analysis of children’s calls to telephone consultation support programmes identified that abuse/violence, mental health, peer and family relationships, and sexual development concerns were frequently raised across countries [54]. Considering the unique capabilities of telephone consultation support programmes, including the fact that they are time sensitive, cost effective and anonymous, it is believed that telephone consultation support programmes are effective tools that can be utilised to provide brief intervention to individuals experiencing suicidal crises [41]. Furthermore, the current telephone consultation support programme was developed to accommodate the unique needs of young individuals in Japan [12]. These unexpected/impulsive analysis results for the municipal telephone consultation support programme are possibly related to an increase in school-aged female suicide mortality caused by school-related motives, providing important information regarding current regional suicide prevention programmes in Japan. A Japanese study using data from “Inochi No Denwa”, which is the largest volunteer-run telephone consultation support organisation with a number of branches throughout Japan, indicated that the rate of suicidal ideation was about 14% [39]. Therefore, the Japanese rate of suicidal ideation in telephone consultation support programmes is smaller than that in the USA (about 30%) [55]. Furthermore, school-aged individuals were more likely to have problems related to school and romance, but the rate of suicidal ideation was lower than 10% [39]. The data suggest that telephone consultation support programme callers do not always have suicidal ideation in Japan. Japanese telephone consultation support programme counsellors should be required to possess the skills to identify individuals at high risk of suicide among the many telephone consultation support programme callers they speak with. In other words, the identification of telephone consultation support programme callers at a high risk of suicide and the development of guidelines for rational responses to these callers remain a challenge in Japan.

The prefectural enlightenment programme was inversely and positively related to male and female suicide mortalities of school-aged population (total), respectively, without affecting suicide mortality caused by school-related motives of school-aged populations. Most of the schools in Japan are prefectural/municipal schools, and school education is mainly managed/operated by the board of education of the prefectures/municipalities [56,57]. This Japanese governmental system seems to be advantageous for policies that concern school-aged individuals; however, it is likely that this system does not generate effective collaboration between the education system and suicide prevention programmes, since the regional suicide prevention programme is mainly organised by the Health/Welfare Divisions of prefectures/municipalities.

### 4.3. Limitations

Suicide is a more frequent event in the elder population, and suicide mortality is also still high in East Asia countries including Japan, which are aging countries. The present results that governmental financial support contributes to the reduction in suicide mortality should be a meaningful finding. However, there are several limitations to this study. EFECBSC was a fund to support the development of social resources/welfare necessary for regional suicide prevention programmes, not a grant (instead of using up in each year). EFECBSC allows flexible operation according to the development of regional suicide prevention programmes of each regional government, and regional governments were also allowed to execute the amount funding of EFECBSC until 2014. Based on the EFECBSC characteristics, it was not possible to analyse the time-dependent effect of EFECBSC on suicide mortality (correlation between EFECBSC and suicide mortality for each year). Therefore, the present study converted the panel data of prefectural suicide mortality data to cross-section data (EBSMR trends) to analyse the effects of the funding amount of EFECBSC on suicide mortalities. This study seems to use regression analysis without prefectural fixed effects, but the conversion to EBSMR trends led to almost equal to consideration of the prefectural fixed effects. 

The major purpose of this study was to evaluate the cost-effectiveness of EFECBSC on suicide mortality in Japan, and the results suggest that the financial support of the government probably contributed to, at least partially, a reduction in suicide mortality; however, the detailed effects of any factors on the reduction in suicide mortality in Japan using panel data with fixed effects have remained to be clarified. Therefore, to respond to the concerns posed by suicide in aging countries, we shall report further studies which analyse comprehensive suicide prevention and resilience factors using fixed-effect models.

## 5. Conclusions

The present study suggests that governmental financial support, EFECBSC, at least partially, contributes to a reduction in suicide mortality in Japan. The most frequent male and female suicide mortalities caused by health-related motives were inversely related to the prefectural intervention model programme and the municipal development programme of leaders/listeners, respectively. Male suicide mortality caused by economy-related motives was also inversely related to the prefectural intervention model programme, but that of females was not related to any EFECBSC sub-divisions. Surprisingly, female suicide mortalities caused by family- and school-related motives were positively related to the prefectural and municipal telephone consultation support programmes, respectively. The results of our statistical analysis suggest the existence of gender-specific responses, even for suicide caused by the same motives, since the sensitivities of males and females to the EFECBSC sub-divisions are not identical. Furthermore, despite being effective tools, both the prefectural and municipal telephone consultation support programmes probably increased female suicide mortality caused by family- and school-related motives, respectively. However, regional suicide prevention programmes in Japan do seem to contribute to improving suicide mortality, but parts of the suicide prevention programme must be improved, such as the telephone consultation support programme. Therefore, throughout the past decade, the Japanese suicide prevention programmes can be evaluated as having achieved success in reducing male suicide mortality; however, these programmes are possibly ineffective, or have an adverse effect, in regard to the suicide mortalities of female and school-aged populations. In other words, according to our evaluation of the EFECBSC, we should work to improve regional suicide prevention programmes, making them more cost-effective and targeted towards female and school-aged populations in the future.

## Figures and Tables

**Table 1:** Gender ratio of numbers of the six major suicide motives in the 47 prefectures of Japan from 2009 to 2018 (N = 470). Data are the mean ± standard deviation (maximum–minimum) (%).

**Motives for Suicide**	**Male**	**Female**
Total	69.8 ± 3.0 (79.1–59.6)	30.2 ± 3.0 (40.4–20.9)
Health	58.9 ± 5.2 (78.2–40.5)	41.1 ± 5.2 (59.5–21.8)
Economy	89.0 ± 7.1 (100.0–60.0)	11.0 ± 7.1 (40.0–0.0)
Family	64.4 ± 8.0 (92.9–37.5)	35.7 ± 8.0 (62.5–7.1)
Employment	89.0 ± 7.1 (100.0–57.1)	11.0 ± 7.1 (42.9–0.0)
Romance	65.4 ± 17.2 (100.0–0.0)	34.6 ± 17.2 (100.0–0.0)
School	75.0 ± 25.2 (100.0–0.0)	25.1 ± 25.2 (100.0–0.0)

**Table 2:** Effects of the funding for the sub-divisions of the Emergency Fund to Enhance Community-Based Suicide Countermeasures (EFECBSC) on Male+Female, male and female EBSMR trends of suicide mortalities caused by family-, health-, economy- and school-related motives. The red colour sub-divisions of the EFECBSC indicate negative effects on the prevention of suicide mortality. VIF: variance inflation factor.

Male+Female	Adjusted R^2^	F	*p*	Factor		β	*p*	VIF
health	0.087	5.405	0.025	Prefectural	Intervention model	−0.327	0.05	1.000
economy	0.104	6.316	0.016	Prefectural	Intervention model	−0.351	0.016	1.000
**Male**	**Adjusted R^2^**	**F**	***p***	**Factor**		**β**	***p***	**VIF**
health	0.150	5.066	0.010	Prefectural	Intervention model	−0.364	0.05	1.024
				Prefectural	Personal consultation	+0.297	0.05	1.024
economy	0.108	6.551	0.014	Prefectural	Intervention model	−0.356	0.05	1.100
**Female**	**Adjusted R^2^**	**F**	***p***	**Factor**		**β**	***p***	**VIF**
family	0.066	4.228	0.046	Prefectural	Telephone consultation	+0.293	0.05	1.000
health	0.086	5.431	0.025	Municipal	Development leaders/listeners	−0.326	0.05	1.000
school	0.063	4117	0.048	Municipal	Telephone consultation	+0.290	0.05	1.000

**Table 3:** Effects of funding for the sub-divisions of EFECBSC on Male+Female, male and female EBSMR trends of suicide mortality of school-aged population (total: suicide numbers of those younger than 20 years old/population of those younger than 20 years old) and caused by school-related motives (school-related motive: suicide numbers caused by school-related motives/population of those younger than 20 years old). The effects of the amount of funding provided to 10 EFECBSC sub-divisions on the EBSMR trends of Male+Female, males and females were analysed by forward multiple regression analysis. The red colour sub-divisions of the EFECBSC indicate negative effects on the prevention of suicide mortality.

Male+Female	Adjusted R^2^	F	*p*	Factor		β	*p*	VIF
Total	0.167	5.625	0.001	Prefectural	Development leaders/listeners	−0.344	0.05	1.00
				Municipal	Intervention model	−0.301	0.05	1.00
**Male**	**Adjusted R^2^**	**F**	***p***	**Factor**		**β**	***p***	**VIF**
Total	0.109	6.600	0.014	Prefectural	Enlightenment	−0.358	0.05	1.009
**Female**	**Adjusted R^2^**	**F**	***p***	**Factor**		**β**	***p***	**VIF**
Total	0.306	5.766	0.004	Prefectural	Personal consultation	+0.375	0.01	1.031
				Prefectural	Enlightenment	+0.304	0.01	1.045
				Municipal	Telephone consultation	+0.321	0.05	1.059
School-related motives	0.071	4.517	0.039	Municipal	Telephone consultation	+0.302	0.05	1.000

## Data Availability

All data relevant to the study are included in the article or uploaded as supplementary information. All raw data are available to any persons from Japanese National databases in the Statistics Bureau of the Ministry of Internal Affairs and Communications (SBMIAC), Cabinet Office (CAO) and Ministry of Health, Labour and Welfare (MHLW).

## Supplementary Materials

The following are available online at https://www.mdpi.com/1660-4601/18/7/3414/s1. Supplementary Table S1. Effects of the amounts of fund of EFECBSC sub-divisions on Male+Female, male and female EBSMR trends of suicide mortalities caused by health- related motive. Supplementary Table S2. Effects of the amounts of fund of EFECBSC sub-divisions on Male+Female, male and female EBSMR trends of suicide mortalities caused by economy- related motive. Supplementary Table S3. Effects of the amounts of fund of EFECBSC sub-divisions on Male+Female, male and female EBSMR trends of suicide mortalities caused by family-related motive. Supplementary Table S4. Effects of the amounts of fund of EFECBSC sub-divisions on Male+Female, male and female EBSMR trends of suicide mortalities caused by school-related motive. Supplementary Table S5. Effects of the funding amounts of EFECBSC sub-divisions on Male+Female, male and female EBSMR trends of suicide mortalities of school-aged population (total). Supplementary Table S6. Effects of the funding amounts of EFECBSC sub-divisions on Male+Female, male and female EBSMR trends of suicide mortalities caused by school-related motive of school-aged population (school-related motive).
