# Supplementary material for: Analysing the Impacts of Financial Support for Regional Suicide Prevention Programmes on Suicide Mortality Caused by Major Suicide Motives in Japan Using Statistical Government Data
Source: Int J Environ Res Public Health. 2021 Mar 25;18(7):3414. doi: 10.3390/ijerph18073414 (PMC8036759; doi:10.3390/ijerph18073414)
Supplement: Supplementary file 1 [file ijerph-18-03414-s001.pdf]

Supplementary Tables

Supplementary tables indicate the statistical values regarding effects of EFECBSC sub-divisions on EBSMR trends of suicide mortalities caused by six major motives. When the data did not violate the ANOVA (p>0.05), any the values of forward multiple regression are not described. P values of excluded variables by forward multiple regression analysis are also not described.

Supplementary Table S1. Effects of the amounts of fund of EFECBSC sub-divisions on Male+Female, male and female EBSMR trends of suicide mortalities caused by health- related motive.

**Health**	**Male+Female**		**Male**		**Female**	
Adjusted R^2	0.087		0.150		0.086	
F value	5.405		5.066		5.341	
P value	0.025		0.010		0.025	
	β	P	β	P	B	P
Prefecture						
Personal consultation support	0.204		0.297	0.037	-0.061	
Telephone consultation support	0.128		0.105		0.084	
Development leaders/listeners	0.094		-0.053		0.005	
Enlightenment	-0.125		-0.145		-0.153	
Intervention model	-0.327	0.025	-0.364	0.011	-0.256	
Municipal						
Personal consultation support	0.056		0.021		-0.077	
Telephone consultation support	0.105		0.118		0.120	
Development leaders/listeners	-0.185		-0.114		-0.326	0.025
Enlightenment	-0.188		-0.210		-0.163	
Intervention model	0.028		-0.008		-0.087	
Complete unemployment rate	0.025		0.003		0.015	

Supplementary Table S2. Effects of the amounts of fund of EFECBSC sub-divisions on Male+Female, male and female EBSMR trends of suicide mortalities caused by economy- related motive.

**Economy**	**Male+Female**		**Male**		**Female**	
Adjusted R^2	0.104		0.108			
F value	6.316		6.551			
P value	0.016		0.014			
	β	P	β	P	B	P
Prefecture						
Personal consultation support	0.196		0.181		0.174	
Telephone consultation support	0.269		0.258		0.349	
Development leaders/listeners	0.161		0.139		-0.041	
Enlightenment	-0.002		-0.026		-0.245	
Intervention model	-0.351	0.016	-0.356	0.014	0.019	
Municipal						
Personal consultation support	-0.014		-0.012		-0.060	
Telephone consultation support	0.036		0.049		0.020	
Development leaders/listeners	-0.250		-0.253		-0.132	
Enlightenment	-0.170		-0.197		0.158	
Intervention model	-0.076		-0.050		-0.289	
Complete unemployment rate	0.183		0.194		0.223	

Supplementary Table S3. Effects of the amounts of fund of EFECBSC sub-divisions on Male+Female, male and female EBSMR trends of suicide mortalities caused by family-related motive.

**Family**	**Male+Female**		**Male**		**Female**	
Adjusted R^2					0.066	
F value					4.228	
P value					0.046	
	β	P	β	P	B	P
Prefecture						
Personal consultation support	0.051		0.011		0.132	
Telephone consultation support	0.243		0.213		0.293	0.046
Development leaders/listeners	0.031		-0.020		0.108	
Enlightenment	-0.012		-0.091		-0.041	
Intervention model	-0.288		-0.289		0.026	
Municipal						
Personal consultation support	0.275		0.324		0.167	
Telephone consultation support	0.064		0.095		0.057	
Development leaders/listeners	-0.234		-0.259		0.015	
Enlightenment	-0.050		-0.001		-0.014	
Intervention model	-0.017		-0.105		0.020	
Complete unemployment rate	-0.180		-0.062		-0.216	

Supplementary Table S4. Effects of the amounts of fund of EFECBSC sub-divisions on Male+Female, male and female EBSMR trends of suicide mortalities caused by school-related motive.

**School**	**Male+Female**		**Male**		**Female**	
Adjusted R^2					0.063	
F value					4.117	
P value					0.048	
	β	P	β	P	B	P
Prefecture						
Personal consultation support	0.196		0.113		0.105	
Telephone consultation support	-0.066		-0.123		0.123	
Development leaders/listeners	-0.226		-0.100		-0.063	
Enlightenment	-0.213		-0.243		0.001	
Intervention model	-0.079		-0.150		-0.081	
Municipal						
Personal consultation support	0.049		0.061		0.053	
Telephone consultation support	0.144		0.060		0.290	0.048
Development leaders/listeners	0.016		-0.122		-0.012	
Enlightenment	0.455		0.503		0.077	
Intervention model	-0.352		-0.147		-0.026	
Complete unemployment rate	0.108		0.023		0.116	

Supplementary Table S5. Effects of the funding amounts of EFECBSC sub-divisions on Male+Female, male and female EBSMR trends of suicide mortalities of school-aged population (total).

**School-aged population (total)**	**Male+Female**		**Male**		**Female**	
Adjusted R^2	0.167		0.109		0.306	
F value	5.625		6.600		5.766	
P value	0.007		0.014		0.021	
	β	P	β	P	B	P
Prefecture						
Personal consultation support	0.080		-0.108		0.375	0.004
Telephone consultation support	-0.212		-0.132		-0.094	
Development leaders/listeners	-0.344	0.014	-0.262		0.152	
Enlightenment	-0.190		-0.358	0.014	0.304	0.021
Intervention model	-0.088		-0.137		0.115	
Municipal						
Personal consultation support	0.097		-0.082		0.053	
Telephone consultation support	0.035		0.107		0.321	0.014
Development leaders/listeners	0.065		0.033		0.025	
Enlightenment	-0.116		-0.046		-0.302	
Intervention model	-0.301	0.030	-0.218		-0.145	
Complete unemployment rate	0.194		-0.027		0.207	

Supplementary Table S6. Effects of the funding amounts of EFECBSC sub-divisions on Male+Female, male and female EBSMR trends of suicide mortalities caused by school-related motive of school-aged population (school-related motive).

**School-aged population (school-related motive)**	**Male+Female**		**Male**		**Female**	
Adjusted R^2					0.071	
F value					4.517	
P value					0.039	
	β	P	β	P	B	P
Prefecture						
Personal consultation support	0.240		0.140		0.131	
Telephone consultation support	-0.090		-0.130		0.142	
Development leaders/listeners	-0.171		-0.115		-0.048	
Enlightenment	-0.199		-0.252		0.020	
Intervention model	-0.097		-0.123		-0.050	
Municipal						
Personal consultation support	0.005		0.072		0.069	
Telephone consultation support	0.175		0.065		0.302	0.039
Development leaders/listeners	-0.028		-0.135		-0.021	
Enlightenment	0.481		0.523		0.086	
Intervention model	-0.290		-0.163		-0.009	
Complete unemployment rate	0.041		0.019		0.121	
